# Associations between Disability and Infertility among U.S. Reproductive-Aged Women

**DOI:** 10.3390/ijerph18063202

**Published:** 2021-03-19

**Authors:** Sandie Ha, Valerie Martinez

**Affiliations:** Department of Public Health, School of Social Sciences, Humanities and Arts, Health Science Research Institute, University of California, Merced, CA 95343, USA; vmartinez44@ucmerced.edu

**Keywords:** disability, infertility, reproductive health, women’s health

## Abstract

We aim to evaluate the association between self-reported disabilities and infertility and whether disabilities are associated with decreased likelihood of seeking infertility-related care. This US nationally representative cross-sectional analysis includes 3789 non-pregnant women aged 18–49 years without history of hysterectomy or oophorectomy (NHANES, 2013–2018). Disabilities and infertility were both self-reported in personal interviews with trained interviewers. Logistic regression models estimated the adjusted odds ratio (aOR) and 95% confidence intervals for the association between disabilities and infertility and related care seeking. Models adjusted for potential confounders and complex probability sampling. Compared to women without disabilities, women with disabilities (WWD) had higher odds of infertility (aOR: 1.78 (1.31–2.40)). Similar findings were observed for sensory (2.32 (1.52–3.52)) and cognitive disabilities (1.77 (1.28–2.44)). Among women with infertility, WWD were less likely to seek infertility-related care (0.68 (0.32–1.44)) but these estimates were not statistically significant. WWD have increased odds of reporting infertility, and if affected, are less likely to visit a health care provider for this condition. While more research is needed to understand reproductive health issues and needs among WWD, it is important to push for more equitable policies and practices to address the health needs of this underserved population.

## 1. Introduction

Approximately one in four adults in the United States (US) are affected by some form of disability [1]. The prevalence of disability increases with age and is approximately 12% among reproductive age women [2,3]. Despite the significant proportion of US women of reproductive age affected by disability, reproductive health in this population has not received much attention. Women with disabilities (WWD) who express a desire to become pregnant may encounter numerous barriers to reproductive health, including limited access to quality healthcare services and discouraging reactions from family members, healthcare providers, and peers. Research is needed to further understand reproductive health issues and needs for WWD [4,5].

Medical advances and the increased prevalence of non-communicable chronic conditions among reproductive age women are leading to an increase in the population of WWD within this age group. Recent improvements in perinatal care have also led to increased survival of infants who were born prematurely or with serious perinatal outcomes such as spina bifida and cerebral palsy, contributing to increased prevalence of disabilities as these babies reach their reproductive years. Studies have shown that women with and without disabilities report similar desire and intention of becoming pregnant [6]. As such, more WWD are planning to have children today than in the past [7,8,9]. As the population of WWD grows, it is important to explore their reproductive health and related needs.

Reproductive health among WWD is highly understudied; however, some studies have shown that WWD have a significantly higher risk of having adverse pregnancy outcomes compared to women without disabilities [10]. A study using Medical Expenditure Panel Survey annualized data (1996–2007) reported that women with complex activity limitations and social restrictions were 55% times more likely to have miscarriages compared to women without these complications [11]. In another study, which uses data from the Nationwide Inpatient Sample of the Healthcare Cost and Utilization Project (2007–2011), women with intellectual and developmental disabilities had higher risk of having a pregnancy affected by preterm birth, low birth weight, and stillbirth [12]. Similar studies also show that women with intellectual and developmental disabilities had higher risk of early labor, preterm birth, and preeclampsia, and their infants were more likely to have low birth weight, and low Apgar scores [13,14]. A recent report of singleton live birth deliveries in Washington State (1987–2012) found WWD had significantly higher risk of gestational diabetes, preeclampsia, and inadequate prenatal care compared to unaffected women [15]. Their infants were also more likely to be small for gestational age [15].

The link between disability and infertility is also plausible but very few studies have investigated infertility risk among women with and without disabilities. A cross-sectional study using data from the National Survey of Family Growth 2011–2015 shows that women with a self-identified cognitive disability experience significant decreases in fecundity, defined as the probability of conceiving within a menstrual cycle for a woman having regular unprotected intercourse [16]. To our knowledge, there are no other studies that directly investigate whether disability is associated with infertility.

WWD face multiple barriers to reproductive health services. While it is known that preconception care guidelines are generally not adequately met in reproductive age women, WWD generally receive even less preconception care compared to those without disabilities [17,18]. WWD also experience more psychological stress, higher levels of systemic oxidative stress, and higher prevalence of comorbidity, all of which may affect their ability to conceive [19,20,21,22]. In addition, the prevalence of health risk factors such as smoking, alcohol consumption, and higher body mass index is also higher among WWD compared to their counterparts [23], making this a particularly vulnerable group with respect to reproductive health outcomes.

Limited understanding of reproductive health risk among reproductive age WWD has contributed to challenges in designing equitable policies and practices to address the health needs of this population. As a result, this vulnerable population is often overlooked in terms of their reproductive health and related needs [24,25]. According to a report from the National Center for Health Statistics, infertility rates are increasing in the US [26,27]. This is a concerning trend as pregnancy is an important life event for some WWD, and reproductive difficulties can lead to negative mental and emotional health consequences. In addition, if a woman elects medical intervention for infertility, the financial burden is also significant, and more vulnerable populations including WWD may be significantly less likely to have access to care and treatment. Studies examining reproductive healthcare access among WWD who are affected by infertility are limited.

The purpose of this study is two-fold. First, we aim to determine the association between disability and self-reported infertility among a US representative group of reproductive age women. Second, we seek to explore whether disability status affects whether women seek medical attention for infertility. We hypothesize that disability is positively associated with the odds of self-reported infertility, and among those affected with infertility, WWD are less likely to seek medical attention related to their difficulties getting pregnant.

## 2. Materials and Methods

### 2.1. Data and Participants

Data came from the 2013–2014, 2015–2016, and 2017–2018 waves of the National Health and Nutrition Examination Survey (NHANES) [28]. NHANES is a continuous cross-sectional survey administered to a representative sample of approximately 5000 individuals across the US per year. The purpose of this nationally representative survey is to assess the health and nutrition status of US adults and children. NHANES data are sampled using a complex, multistage, probability sampling design to select participants from the non-institutionalized population. To ensure reliable statistics, the survey over-samples persons 60 years and older, African Americans, and Hispanics. It combines in-person interviews, physical examinations, and laboratory tests. Data are released in two-year cycles. Since data obtained for this study are publicly available and are completely deidentified, informed consent and institutional review board approval were not necessary.

There was a total of 29,400 participants from the three data cycles. Although the female reproductive age ranges from 15 to 49, the reproductive health questionnaire, which includes information on infertility, was only assessed for women older than 18. After excluding 14,452 males, 5630 females <18 years old, 4428 females above 49 years old, 736 without a response to the infertility question, 223 with a history of hysterectomy, 2 with a history of oophorectomy, and 140 who are currently pregnant (all mutually exclusive), we ended up with 3789 participants in the final analyses (Figure S1).

### 2.2. Exposure Assessment

The main exposure of interest is self-reported disability, which was assessed in the disability-specific questionnaire of the NHANES. This questionnaire was performed through computer-assisted personal interviews conducted by trained staff in participants’ homes. Interpreters were used if participants did not speak English or Spanish. Participants were asked if they were deaf or had serious difficulty hearing; blind or had serious difficulty seeing even with corrections; had serious difficulty concentrating, remembering, or making decisions because of a physical/mental/emotional condition; had serious difficulty walking or climbing stairs; had difficult dressing or bathing; or had difficult doing errands alone (Table S1). Participants were classified as having a disability if they answered yes to any of the questions above. As different types of disability may affect infertility and reproductive care access differently, it is also important to explore the effects of specific disability types. Thus, disabilities were also classified into five types: physical, sensory, cognition, self-care, and independent living (Table S1). Physical disabilities included having serious difficulty walking or climbing stairs. Sensory disabilities include deafness, blindness, or serious difficulty with hearing or seeing even with correction. Cognitive disabilities include serious difficulty with concentrating, remembering, or making decisions. Self-care disabilities including difficulty with dressing or bathing. Lastly, independent living disabilities include difficulty running errands alone such as visiting a doctor’s office or shopping (Table S1). All disabilities were dichotomized as present or absent. The questionnaire did not collect information on the severity or start time of disabilities.

### 2.3. Outcome Assessment

The main outcomes of interest are self-reported infertility and whether women sought medical attention for being unable to become pregnant. All female participants ages 18 or over were asked if they had “ever attempted to become pregnant over a period of at least a year without becoming pregnant”, and “ever been to a doctor or other medical provider because [they have] been unable to become pregnant”. These questions were administered in the reproductive health questionnaire using computer-assisted personal interview with trained interviewers. Women were classified as having self-reported infertility if they answered “yes” to the first question. Similarly, if they answered affirmatively for the second question, they were classified as having sought reproductive healthcare for infertility.

### 2.4. Covariates

We considered several potential covariates, which were obtained from computer-assisted personal interviews or medical examination by highly trained medical personnel. These variables included age, marital status, education, race/ethnicity, insurance status, smoking, gravidity, income, general health status, and chronic diseases including cancer, cardiovascular diseases, thyroid diseases, and arthritis. In addition, body mass index was obtained from physical examination. Covariates that were included in the final models were selected a priori based on a directed acyclic graph (DAG), an important tool commonly used in epidemiologic research to conceptualize variables that are relevant for the relationship between an exposure and an outcome [29]. Different DAGs were considered for different exposure and outcome combinations, but the current literature suggests two DAGs—one for self-report infertility and the other for seeking infertility treatment (Figure S2).

### 2.5. Statistical Analyses

Chi-square, Fisher’s exact, and *t*-tests were used to compare characteristics between (a) women who reported infertility vs. those who did not, and (b) women who sought reproductive health care services for infertility vs. those who did not among those with infertility. Weighted logistic regression models were used to obtain the odds ratio (OR) and 95% confidence intervals (CI) for the associations between disability and self-reported disability and infertility as well as care-seeking behavior for infertility-related problems among those affected. We ran two different models including (a) an unadjusted model, and (b) a DAG-based model where only variables that met confounding criteria were included (Figure S2). For all models, the comparison group was women who did not have any disability. All statistical analyses were performed using SAS 9.4 (Cary, NC, USA) and accounted for the complex survey design.

## 3. Results

The final analyses included a total of 3789 non-pregnant women ages from 18 to 49 who do not have a history of hysterectomy or oophorectomy. Table 1 presents the characteristics of the study participants by infertility status. The estimated prevalence of self-reported infertility in the study population was 11.5% (95% CI: 10.1–12.9). The prevalence of any disability is 15.2% (95% CI: 13.3–17.2). Cognitive disabilities were the most prevalent (9.0%, 95% CI: 7.8–10.3), followed by independent living (5.2%; 95% CI: 4.2–6.3), physical (4.7%; 95% CI: 3.7–5.8), sensory (4.6%, 95% CI: 3.6–5.5), and self-care disability (1.4%, 95% CI: 1–1.9) (Table 1). Self-reported infertility was significantly more prevalent among women who had any disabilities compared to those without (16.6% vs. 10.6%). Self-reported infertility was also more prevalent among women who were older, married/cohabiting, had higher income to poverty ratio, parous, underweight or obese, former or current smoker, had worse general health, or had chronic conditions such as thyroid problems, arthritis, or cancer. Characteristics of participants by disability status are also presented in Table S2. In general, disabilities were more prevalent among women of Black, Hispanic, and Other races/ethnicities, those with less education, divorced/single/widowed, had no insurance, poorer, parous, underweight or obese, current or former smoker, had poorer health status, and had chronic diseases such as arthritis, thyroid problems, cardiovascular diseases, or cancer.

Table 2 presents characteristics of women who reported infertility by whether they sought reproductive care for infertility. Among women affected by infertility, the proportion who sought reproductive care services for infertility was lower among those with disability compared to those without (49.0% vs. 61.7%), but these differences were not statistically significant. A consistent pattern was observed for physical, sensory, and cognitive disabilities but not for self-care and independent living disability. Those who sought reproductive health services for infertility were more likely to be older, Asian, non-Hispanic white, or other races/ethnicity, had at least some college education, were married/cohabitating, had insurance, had higher income to poverty ratio, parous, normal weight, did not smoke, or had thyroid problems, cardiovascular diseases, or cancer.

**Figure 1:**
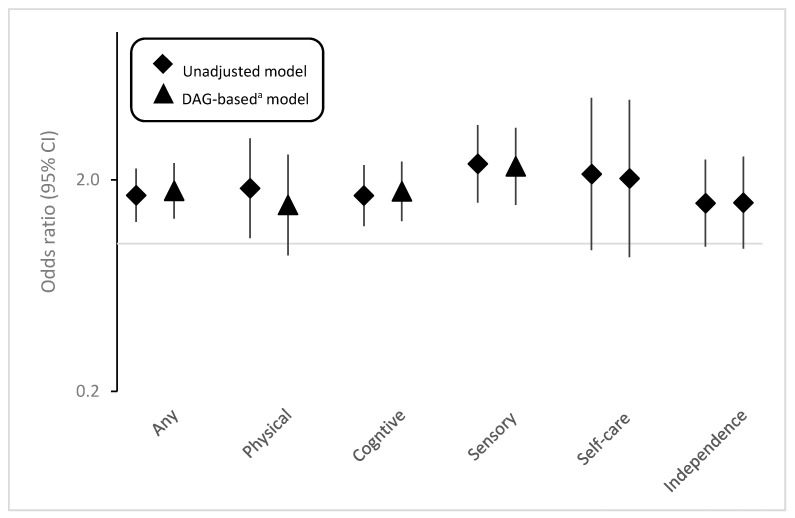
Associations between disability and self-report infertility, NHANES 2013–2018. ^a^ DAG-based model adjusted for age, race, and education. The *Y*-axis is displayed on the logarithmic scale.

Having any disability was associated with increased odds of having self-reported infertility in both unadjusted and adjusted models (Figure 1, Table S3). After adjusting for confounders, compared to those without disability, WWD had 78% increased odds of having self-reported infertility (aOR:1.78, 95% CI: 1.31–2.40). Disabilities related to sensory functions appeared to have the strongest association with infertility (aOR: 2.32, 95% CI: 1.52–3.52), followed by cognitive disabilities (aOR: 1.77, 95% CI: 1.28–2.44). For other types of disabilities (i.e., physical, self-care, independent living), the associations with infertility were positive but not statistically significant (Figure 1, Table S3).

**Figure 2:**
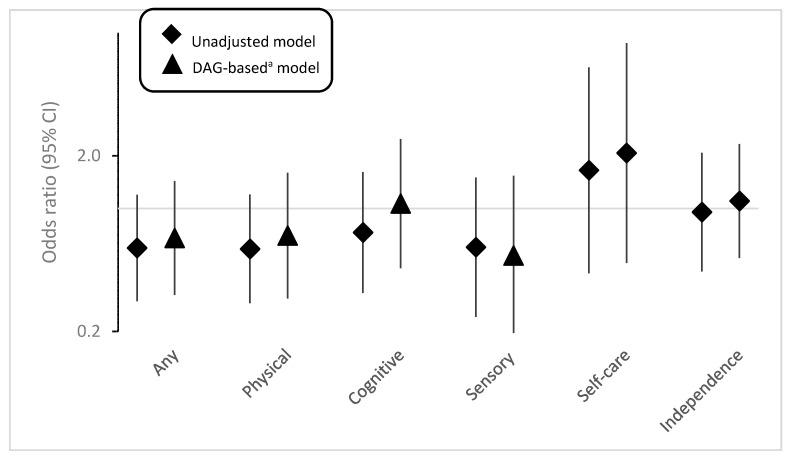
Associations between disability and infertility-related doctor visit among those reported infertility. ^a^ DAG-based model adjusted for age, race, and education. The *Y*-axis is displayed on the logarithmic scale.

Among those who had infertility, having disabilities appeared to be generally related to lower odds of seeking reproductive healthcare for infertility. However, these associations are not statistically significant (Figure 2, Table S4) and may be at least partially related to lack of power due to low sample size. There were positive associations between cognition, self-care, and independent living disability with reproductive care seeking, but these associations were also not statistically significant.

## 4. Discussion

The purpose of this study was to examine the association between disability and self-reported infertility in a nationally representative sample of US reproductive age women aged 18 to 49 with no history of hysterectomy or oophorectomy. We further evaluated, among those who reported infertility, whether disability status is related to seeking reproductive healthcare services for infertility. Results generally suggest that WWD had significantly higher odds of having self-reported infertility after adjusting for confounders. In addition, among those who reported infertility, WWD appeared to have lower odds of seeking reproductive healthcare services for this condition, although these estimates were not statistically significant due to the small sample size.

Reproductive health among WWD is an understudied area of research. Empirical data related to infertility among WWD remain very limited. Nevertheless, our results are consistent with a recent, and, to our knowledge, the only existing study that investigates the relationship between disabilities and infertility. Zhang et al. compared self-reported time-to-pregnancy among 383 women aged 18–44 years with and without disabilities in the National Survey of Family Growth who were attempting pregnancy [16]. Their findings suggested that women with self-reported cognitive disabilities had significantly lower fecundity (i.e., took longer to get pregnant) than women without disabilities (fecundability hazard ratio: 0.56, 95% CI: 0.30–0.88). Our data also suggest that women with cognitive disabilities had an approximately 58% increased odds of infertility. We also found positive associations with sensory and physical disabilities while Zhang et al. did not. This discrepancy could be due to the smaller sample size in Zhang et al., the difference in age range, and the difference in how infertility was operationalized.

Based on prior research and the results of this study, several broad categories of unmet reproductive health needs for WWD can be identified. These include (a) addressing disparities with respect to socio-behavioral determinants of health, (b) clinician knowledge and attitudes, (c) accessibility of health care facilities and equipment, and (d) more research and data to guide policy and practice. First, although the exact causal mechanism linking disabilities and infertility is unclear and may vary by individual, a few pathways may explain this association. There are significant disparities in terms of comorbidities, access to quality healthcare, and behavioral and social determinants of health between WWD and those without disabilities [30]. People with disabilities consistently report higher rates of obesity, lack of physical activity, and smoking [31]. They also have higher incidence of diabetes and cardiovascular diseases [32,33]. Mental distress such as depression or anxiety is also a common concern for this population, who are also more likely to report receiving inadequate social and emotional support [34,35]. Despite the higher rates of chronic diseases, adults with disabilities are significantly less likely to receive preventive care [36,37], which puts them at greater risk of unfavorable reproductive health outcomes including infertility. As a result, there is an urgent need to further understand and address disparities in socio-behavioral risk factors that could be contributing to higher risk of infertility and other reproductive complications among WWD. Our analyses adjusted for some of these risk factors, but the associations remained robust, suggesting that the effects of disability on infertility may be explained by reasons beyond comorbidities and behavioral risk factors.

In healthcare settings, persons with disabilities are three times as likely to be denied care and four times as likely to be treated negatively by health care providers [38]. They are also more likely than their counterparts to report that medical equipment does not adequately meet their needs and that their doctors do not listen to them, treat them with respect, take enough time, involve them in treatment decisions, or explain treatments properly [38,39]. The lack of adequate medical training with respect to patients with disabilities have contributed to increased negative medical encounters for WWD and, ultimately, inadequate preconception care even among those with medical insurance. A survey sent to a random sample of 1000 OBGYN physicians across the US reported that while most respondents indicated feeling “somewhat” (57.5%) or “very” (21.9%) aware of the special healthcare needs of WWD, only 17.2% received any information or training on the provision of healthcare to WWD [40]. A large survey of medical school deans suggested that medical students receive limited training in the care of people with disabilities and therefore may not be able to adequately meet the competencies [41]. Studies have consistently shown that healthcare providers often have inaccurate assumptions and beliefs about WWD’s decision making ability, sexual and reproductive interests, pregnancy risks, probability of successful treatment, and parenting ability [24]. As the global prevalence of disabilities increases, there is a critical need for more training among healthcare providers to address negative attitudes and assumptions about the sexuality, childbearing desires, and parenting ability of WWD. In addition, despite the passage of the American Disabilities Act Standards for Accessible Design, studies have shown that forty-four percent of gynecology practices are inaccessible to WWD [42]. More recently, the US Access Board has established standards for accessible equipment including adjustable-height examination tables. Availability of such equipment would greatly improve access to general and reproductive health care for WWD.

A nationally representative study among 10,782 US women aged 15–44 shows that women with and without disabilities have similar attitudes toward motherhood. Among women without children, women with and without disabilities were equally likely to want a child and equally likely to intend to have one [6]. The fact that many WWD are sexually active and wish to become mothers suggests a clear and critical need to better understand the entire spectrum of their reproductive healthcare needs, including fertility status [43,44,45]. As existing research on reproductive health among WWD focuses mostly on unintended pregnancies, more research and data to understand reproductive health and related needs among this vulnerable population are critical to guide policy and practice. Furthermore, studies exploring reproductive health among WWD are generally limited from cross-sectional data, which lends limited ability to explore causal pathways. Prospective cohorts are needed to longitudinally capture more details about the nature of disability, risk factors, reproductive health as well as family health over time.

The study has several limitations. First, the cross-sectional nature of the NHANES does not allow inferences regarding the temporal relationship between disability and infertility. In other words, reverse causation is possible in some women as we do not have information on the timing of infertility and disability. Second, both disability and infertility status were self-reported, leading to potential misclassification, especially with infertility status. Studies have suggested that self-reported infertility is a useful measure for quantifying population-level burden of fertility with high specificity (95%) and sensitivity (70%) when validated against medical records [46]. This means that some participants with infertility may have incorrectly reported that they did not have the condition. Since we do not have evidence of differential misclassification between those with and without disability, we expect our results to be biased towards the null and therefore conservative. Due to small sample size, we were unable to evaluate the effects of different combinations of disabilities, nor perform further analyses evaluating potentially moderating effects of important risk factors such as body mass index and other chronic diseases.

Despite limitations, our study is nationally representative and is one of the few studies to examine the relationship between disability and infertility. To our knowledge, it is the second study addressing this question. Large prospective studies are still needed to confirm these findings and to further understand reproductive health among WWD. Meanwhile, studies that evaluate adverse pregnancy outcomes among WWD using live births should consider methods (e.g., inverse probability weighting) to address the fact that women with disabilities are systematically excluded because they are less likely to become pregnant.

## 5. Conclusions

In a US nationally representative sample, reproductive age women with disabilities had significantly higher odds of having self-reported infertility. In addition, women with disabilities appeared less likely to seek medical attention for their infertility, although estimates were unstable due to low sample size. Multiple disparities related to access to care, chronic health conditions, and socio-behavioral determinants of health may explain the observed associations. Given similar desires to have children among WWD and those without disabilities, there is a critical need for more training among healthcare providers to address negative attitudes and assumptions about the sexuality, childbearing desires, and parenting ability of WWD.

## Figures and Tables

**Table 1:** Characteristics of study participants by fertility status, NHANES 2013–2018 (unweighted *n* = 3789).

Characteristics	All N (% CI) ^a^		Infertility ^a^		No Infertility		*p* ^b^
	N = 3789	% and CI	*n* = 402	% and CI 11.5 (10.1–12.9)	*n* = 3387	% and CI 88.5 (87.1–89.9)	
Any disability							0.002
No	3170	84.8 (82.8–86.7)	304	10.6 (9.1–12.0)	2866	89.4 (88.0–90.9)	
Yes	619	15.2 (13.3–17.2)	98	16.6 (13.2–20.1)	521	83.4 (79.9–86.9)	
Physical disability							0.087
No	3586	95.3 (94.2–96.3)	368	11.2 (9.7–12.6)	3218	88.8 (87.4–90.3)	
Yes	203	4.7 (3.7–5.8)	34	17.7 (10.1–25.4)	169	82.3 (74.6–89.9)	
Sensory disability							0.002
No	3596	95.4 (94.5–96.4)	363	11.0 (9.5–12.4)	3233	89.0 (87.6–90.5)	
Yes	193	4.6 (3.6–5.5)	39	21.9 (15.5–28.4)	154	78.1 (71.6–84.5)	
Cognitive disability							0.014
No	3433	91.0 (89.7–92.2)	347	11.0 (9.6–12.4)	3086	89.0 (87.6–90.4)	
Yes	356	9.0 (7.8–10.3)	55	16.6 (12.3–20.9)	301	83.4 (79.1–87.7)	
Self-care disability							0.164
No	3725	98.6 (98.1–99)	390	11.4 (9.9–12.8)	3335	88.6 (87.2–90.1)	
Yes	64	1.4 (1.0–1.9)	12	20.1 (7.1–33.1)	52	79.9 (66.9–92.9)	
Independent living disability							0.180
No	3591	94.8 (93.7–95.8)	376	11.3 (9.8–12.7)	3215	88.7 (87.3–90.2)	
Yes	198	5.2 (4.2–6.3)	26	15.5 (9.5–21.4)	172	84.5 (78.6–90.5)	
Age (years, mean, CI)	-	33.1 (32.6–33.5)	-	36.9 (35.9–37.9)	-	32.6 (32.1–33.0)	<0.0001
Race							0.303
Non-Hispanic White	1224	57 (52.2–61.7)	152	12.4 (10–14.8)	1072	87.6 (85.2–90.0)	
Non-Hispanic Black	839	13.3 (10.7–16.0)	88	11.1 (8.9–13.4)	751	88.9 (86.6–91.1)	
Hispanic	1058	19.4 (15.8–22.9)	97	9.9 (7.9–11.9)	961	90.1 (88.1–92.1)	
Non-Hispanic Asian	486	6.1 (4.9–7.2)	45	9.0 (6.8–11.2)	441	91.0 (88.8–93.2)	
Others	182	4.3 (3.5–5.0)	20	10.8 (5–16.7)	162	89.2 (83.3–95.0)	
Education							<0.0001
High school or less	1176	28.3 (25.2–31.5)	133	12.3 (9.5–15.0)	1043	87.7 (85.0–90.5)	
At least some college	2291	66.9 (63.6–70.2)	265	11.9 (10.3–13.6)	2026	88.1 (86.4–89.7)	
Unknown	322	4.8 (3.9–5.6)	4	0.6 (0–1.2)	318	99.4 (98.8–100)	
Marital status							<0.0001
Divorced/single/widow	1414	37.2 (34.6–39.9)	109	7.1 (5.7–8.5)	1305	92.9 (91.5–94.3)	
Married/cohabitating	1958	56.5 (53.7–59.3)	286	15.5 (13.3–17.7)	1672	84.5 (82.3–86.7)	
Unknown	417	6.3 (5.3–7.3)	7	1.2 (0.1–2.2)	410	98.8 (97.8–99.9)	
Insurance							0.472
No	852	18.1 (16.0–20.1)	96	12.9 (10.1–15.8)	756	87.1 (84.2–89.9)	
Yes	2931	81.8 (79.7–83.9)	305	11.2 (9.7–12.6)	2626	88.8 (87.4–90.3)	
Unknown	6	0.1 (0–0.3)	1	11.7 (0–35.2)	5	88.3 (64.8–100)	
Income to poverty ratio							0.028
<1	1230	24.7 (22.3–27.2)	107	9.1 (6.9–11.3)	1123	90.9 (88.7–93.1)	
1–2	905	20.4 (18.5–22.3)	94	10.8 (8.4–13.2)	811	89.2 (86.8–91.6)	
>2	1654	54.9 (51.7–58.1)	201	12.8 (10.8–14.8)	1453	87.2 (85.2–89.2)	
Parity							<0.0001
Nulliparous	873	28.1 (25.7–30.6)	58	5.5 (3.9–7.0)	815	94.5 (93.0–96.1)	
Parous	2498	65.5 (63.2–67.9)	336	15.0 (13.1–16.9)	2162	85.0 (83.1–86.9)	
Unknown	418	6.3 (5.3–7.3)	8	1.4 (0.3–2.4)	410	98.6 (97.6–99.7)	
Body Mass Index Categories							0.0003
Underweight	117	2.3 (1.7–2.9)	13	14.2 (5.2–23.3)	104	85.8 (76.7–94.8)	
Normal weight	1296	36.1 (33.3–38.9)	111	8.7 (7–10.4)	1185	91.3 (89.6–93.0)	
Overweight	895	24 (22.3–25.7)	67	8.3 (5.7–10.8)	828	91.7 (89.2–94.3)	
Obese	1481	37.6 (35.3–39.9)	211	16.0(13.1–18.9)	1270	84.0 (81.1–86.9)	
Smoking status							0.045
Never	2773	69.6 (66.8–72.3)	259	10.4 (8.9–11.9)	2514	89.6 (88.1–91.1)	
Ever	394	12.8 (10.8–14.7)	60	14.9 (11.1–18.7)	334	85.1 (81.3–88.9)	
Current	622	17.7 (15.7–19.7)	83	13.3 (9.8–16.7)	539	86.7 (83.3–90.2)	
Health status							0.039
Good or excellent	3009	83.8 (82–85.6)	303	10.9 (9.4–12.3)	2706	89.1 (87.7–90.6)	
Poor, fair, unsure	780	16.2 (14.4–18)	99	14.7 (11.2–18.2)	681	85.3 (81.8–88.8)	
Arthritis							<0.0001
No	3006	83.6 (81.7–85.5)	333	11.4 (9.9–13.0)	2673	88.6 (87.0–90.1)	
Yes	360	10.0 (8.5–11.4)	60	18.5 (13.2–23.7)	300	81.5 (76.3–86.8)	
Unknown	423	6.4 (5.4–7.4)	9	1.5 (0.6–2.4)	414	98.5 (97.6–99.4)	
Thyroid problems							<0.0001
No	3068	84.6 (83.0–86.3)	345	11.7 (10.4–13.0)	2723	88.3 (87.0–89.6)	
Yes	299	8.9 (7.6–10.1)	50	17.0 (10.5–23.5)	249	83.0 (76.5–89.5)	
Unknown	422	6.5 (5.4–7.6)	7	1.1 (0.1–2.1)	415	98.9 (97.9–99.9)	
Cardiovascular diseases							0.105
No	3710	98.2 (97.8–98.7)	389	11.3 (10.0–12.6)	3321	88.7 (87.4–90)	
Yes	79	1.8 (1.3–2.2)	13	21.0 (8.6–33.5)	66	79.0 (66.5–91.4)	
Cancer							<0.0001
No	3276	90.4 (89.2–91.7)	374	11.6 (10.1–13.2)	2902	88.4 (86.8–89.9)	
Yes	97	3.3 (2.6–4.1)	21	26.6 (15.8–37.4)	76	73.4 (62.6–84.2)	
Unknown	416	6.3 (5.3–7.3)	7	1.2 (0.1–2.2)	409	98.8 (97.8–99.9)	

^a^ The sample size (*n*) is unweighted but the % is accounted for complex sampling design. ^b^ *p*-values were obtained using chi-square test for categorical variables, and *t*-tests for continuous variables, all were accounted for complex sampling design.

**Table 2:** Characteristics of affected participants who had a doctor visit for infertility (*n* = 402).

Characteristics	Sought Reproductive Healthcare		Did not Seek Reproductive Healthcare		*p* ^b^
	*n* ^a^	% and 95% CI	*n*	% and CI	
	219	58.9 (54.1–63.7)	183	41.1 (36.3–45.9)	
Any disability					0.154
No	170	61.7 (55.7–67.6)	134	38.3 (32.4–44.3)	
Yes	49	49.0 (34.7–63.3)	49	51 (36.7–65.3)	
Physical disability					0.201
No	202	59.7 (54.8–64.6)	166	40.3 (35.4–45.2)	
Yes	17	48.7 (32.5–64.9)	17	51.3 (35.1–67.5)	
Sensory disability					0.353
No	198	59.8 (54.8–64.9)	165	40.2 (35.1–45.2)	
Yes	21	49.3 (28.9–69.6)	18	50.7 (30.4–71.1)	
Cognition disability					0.565
No	191	59.6 (54–65.2)	156	40.4 (34.8–46)	
Yes	28	54.1 (37.7–70.4)	27	45.9 (29.6–62.3)	
Self-care disability					0.326
No	211	58.5 (53.6–63.4)	179	41.5 (36.6–46.4)	
Yes	8	72.7 (46.0.–99.3)	4	27.3 (0.7–54.0)	
Independent living disability					0.840
No	203	58.8 (53.7–63.8)	173	41.2 (36.2–46.3)	
Yes	16	60.6 (43.8–77.4)	10	39.4 (22.6–56.2)	
Age (years, mean, CI)		38.4 (37.4–39.3)		34.7 (33.3–36.1)	<0.0001
Race					
Non-Hispanic White	88	62.1 (56.4–67.8)	64	37.9 (32.2–43.6)	0.001
Non-Hispanic Black	36	42.6 (30.5–54.6)	52	57.4 (45.4–69.5)	
Hispanic	44	48.3 (35.7–60.8)	53	51.7 (39.2–64.3)	
Non-Hispanic Asian	38	84.0 (71.9–96.1)	7	16.0 (3.9–28.1)	
Others	13	76.9 (59.5–94.3)	7	23.1 (5.7–40.5)	
Education					<0.0001
High school or less	53	39.0 (29.9–48.0)	80	61.0 (52–70.1)	
At least some college	165	67.7 (62.3–73.1)	100	32.3 (26.9–37.7)	
Unknown	1	20.9 (0–56.7)	3	79.1 (43.3–100)	
Marital status					0.002
Divorced/single/widow	45	47.3 (38.9–55.7)	64	52.7 (44.3–61.1)	
Married/cohabitating	173	62.8 (56.7–68.9)	113	37.2 (31.1–43.3)	
Unknown	1	7.9 (0–23.4)	6	92.1 (76.6–100)	
Insurance					
No	36	36.5 (29.1–43.9)	60	63.5 (56.1–70.9)	
Yes	183	64.7 (59.2–70.3)	122	35.3 (29.7–40.8)	
Unknown	0	0	1	100 (100–100)	
Income to poverty ratio					<0.0001
<1	42	42.0 (30.4–53.7)	65	58.0 (46.3–69.6)	
1–2	41	39.2 (29.1–49.2)	53	60.8 (50.8–70.9)	
>2	136	70.5 (64.8–76.1)	65	29.5 (23.9–35.2)	
Gravidity					0.014
Nulliparous	27	50.0 (34.4–65.5)	31	50.0 (34.5–65.6)	
Parous	191	60.7 (55.7–65.8)	145	39.3 (34.2–44.3)	
Unknown	1	6.7 (0–21.7)	7	93.3 (78.3–100)	
Body Mass Index Categories					0.718
Underweight	5	59.6 (28.6–90.6)	8	40.4 (9.4–71.4)	
Normal weight	68	64.9 (54–75.7)	43	35.1 (24.3–46.0)	
Overweight	35	55.5 (42.3–68.8)	32	44.5 (31.2–57.7)	
Obese	111	56.8 (49.2–64.4)	100	43.2 (35.6–50.8)	
Smoking status					0.006
Never	154	64.0 (57.7–70.4)	105	36.0 (29.6–42.3)	
Ever	36	65.1 (51.8–78.3)	24	34.9 (21.7–48.2)	
Current	29	38.0 (26.5–49.4)	54	62.0 (50.6–73.5)	
Health status					0.065
Good or excellent	172	61.8 (55.9–67.7)	131	38.2 (32.3–44.1)	
Poor, fair, unsure	47	47.9 (37.2–58.6)	52	52.1 (41.4–62.8)	
Arthritis					0.254
No	185	60.1 (54.5–65.7)	148	39.9 (34.3–45.5)	
Yes	31	53.9 (40.1–67.7)	29	46.1 (32.3–59.9)	
Unknown	3	30.9 (0–68.0)	6	69.1 (32–100)	
Thyroid problems					0.004
No	181	56.6 (51.8–61.5)	164	43.4 (38.5–48.2)	
Yes	37	76.2 (59.4–93.0)	13	23.8 (7–40.6)	
Unknown	1	7.9 (0–23.4)	6	92.1 (76.6–100)	
CVD					0.642
No	211	58.7 (53.8–63.6)	178	41.3 (36.4–46.2)	
Yes	8	65.1 (39.8–90.4)	5	34.9 (9.6–60.2)	
Cancer					0.015
No	203	58.2 (53.1–63.4)	171	41.8 (36.6–46.9)	
Yes	15	70.7 (48.1–93.3)	6	29.3 (6.7–51.9)	
Unknown	1	7.9 (0–23.4)	6	92.1 (76.6–100)	

^a^ The sample size (*n*) is unweighted but the % is accounted for complex sampling design. ^b^ *p*-values were obtained using chi-square test and Fisher’s exact tests for categorical variables, and *t*-tests for continuous variables, all were accounted for complex sampling design.

## Data Availability

The NHANES data used in this study can be found at https://wwwn.cdc.gov/nchs/nhanes/Default.aspx (accessed on 18 March 2021).

## Supplementary Materials

The following are available online at https://www.mdpi.com/1660-4601/18/6/3202/s1, Figure S1: Study sample selection, NHANES (2013–2018), Figure S2: Directed acyclic graph to identify potential confounders for the relationship between (a) disabilities and infertility and (b) disabilities and seeking infertility treatment, Table S1: Disability definition, Table S2: Characteristics of study participants by disability status, NHANES 2013–2018, Table S3: Associations between disability and infertility, NHANES 2013–2018, Table S4: Associations between disability and infertility-related doctor visit among those reported infertility.
