# Supplementary material for: Associations between Disability and Infertility among U.S. Reproductive-Aged Women
Source: Int J Environ Res Public Health. 2021 Mar 19;18(6):3202. doi: 10.3390/ijerph18063202 (PMC8003727; doi:10.3390/ijerph18063202)
Supplement: Supplementary file 1 [file ijerph-18-03202-s001.pdf]

International Journal of
Environmental Research and Public Health

MDPI

Supplementary Table S1. Disability definition.

Type of disability	National Health and Nutrition and Examination Survey (NHANE) questions
Sensory	Are you deaf or have serious difficulty hearing?
	Are you blind or have serious difficulty seeing even when wearing glasses?
Cognition	Because of a physical, mental, or emotional condition, do you have serious difficulty concentrating, remembering, or making decisions?
Physical	Do you have serious difficulty walking or climbing stairs?
Self-care	Do you have difficulty dressing or bathing?
Independent living	Because of a physical, mental, or emotional condition, do you have difficulty doing errands alone such as visiting a doctor's office or shopping?
Any	Any of the above disability

Supplementary Table S2. Characteristics of study participants by disability status, NHANES 2013–2018.

Characteristics	Disability		No Disability		p [b]
	n = 619	% and CI **15.2 (13.3–17.2)**	n = 3170	% and CI **84.8 (82.8–86.7)**	
Age (years, mean, SD)		33.1(32.7–33.6)		32.6(31.5–33.8)	0.4324
Race/ethnicity					0.0383
Non-Hispanic (NH) White	227	14.9 (12.1–17.7)	997	85.1 (82.3–87.9)	
NH Black	133	15.1 (12.1–18.2)	706	84.9 (81.8–87.9)	
Hispanic	171	16 (13.2–18.8)	887	84 (81.2–86.8)	
NH Asian	45	10 (6.8–13.1)	441	90 (86.9–93.2)	
Others	43	24 (14.4–33.5)	139	76 (66.5–85.6)	
Education					<0.0001
High school or less	292	25.4 (21–29.9)	884	74.6 (70.1–79)	
At least some college	271	10.6 (8.9–12.3)	2020	89.4 (87.7–91.1)	
Unknown	56	19.6 (12.4–26.7)	266	80.4 (73.3–87.6)	
Marital status					0.0277
Divorced/single/widow	270	17.8 (14.3–21.2)	1144	82.2 (78.8–85.7)	
Married/co-habitating	278	13.3 (11.2–15.4)	1680	86.7 (84.6–88.8)	
Unknown	71	17.9 (12.5–23.3)	346	82.1 (76.7–87.5)	
Insurance					
No	168	21 (16.1–25.9)	684	79 (74.1–83.9)	
Yes	451	14 (12.2–15.8)	2480	86 (84.2–87.8)	
Unknown	0	0	6	100 (100–100)	
Income to poverty ratio					<0.0001
<1	277	24.6 (21.1–28.1)	953	75.4 (71.9–78.9)	
1–2	177	19.8 (16.5–23.1)	728	80.2 (76.9–83.5)	
>2	165	9.3 (7.1–11.5)	1489	90.7 (88.5–92.9)	
Gravidity					0.0145
Nulliparous	107	11.5 (8.3–14.6)	766	88.5 (85.4–91.7)	
Parous	441	16.6 (14.4–18.9)	2057	83.4 (81.1–85.6)	
Unknown	71	17.8 (12.4–23.2)	347	82.2 (76.8–87.6)	
Body mass index Categories					<.0001
Underweight	29	28.1 (17.6–38.6)	88	71.9 (61.4–82.4)	
Normal weight	150	11.3 (8.4–14.3)	1146	88.7 (85.7–91.6)	
Overweight	124	11.7 (8.7–14.7)	771	88.3 (85.3–91.3)	
Obese	316	20.4 (17.9–23)	1165	79.6 (77–82.1)	
Smoking status					<0.0001
Never	359	11.9 (10.1–13.7)	2414	88.1 (86.3–89.9)	
Ever	81	16.1 (12.2–20.1)	313	83.9 (79.9–87.8)	
Current	179	27.9 (22.1–33.7)	443	72.1 (66.3–77.9)	
Health status					<0.0001
Good or excellent	350	11.1 (9.4–12.9)	2659	88.9 (87.1–90.6)	
Poor, fair, unsure	269	36.5 (31.9–41.1)	511	63.5 (58.9–68.1)	
Arthritis					<0.0001
No	398	12.4 (10.5–14.4)	2608	87.6 (85.6–89.5)	
Yes	144	35.8 (29.4–42.2)	216	64.2 (57.8–70.6)	
Unknown	77	19.8 (14.5–25.1)	346	80.2 (74.9–85.5)	

Thyroid problems					0.0349
No	474	14.5 (12.4–16.6)	2594	85.5 (83.4–87.6)	
Yes	72	19.9 (14.6–25.3)	227	80.1 (74.7–85.4)	
Unknown	73	18.6 (12.8–24.3)	349	81.4 (75.7–87.2)	
Cardiovascular diseases					<0.0001
No	570	14.4 (12.3–16.4)	3140	85.6 (83.6–87.7)	
Yes	49	62.9 (50.3–75.6)	30	37.1 (24.4–49.7)	
Cancer					0.0322
No	515	14.6 (12.6–16.6)	2761	85.4 (83.4–87.4)	
Yes	33	26.7 (16.4–37)	64	73.3 (63–83.6)	
Unknown	71	17.9 (12.5–23.4)	345	82.1 (76.6–87.5)	
Data cycle					0.1804
2013–2014	204	14.1 (11.5–16.7)	1158	85.9 (83.3–88.5)	
2015–2016	190	13.3 (10.7–16)	1073	86.7 (84–89.3)	
2017–2018	225	18.2 (13.9–22.5)	939	81.8 (77.5–86.1)	

Supplementary Table S3. Associations between disability and infertility, NHANES 2013–2018.

Disability	Odds Ratio (95% CI)	
	**Unadjusted**	**DAG-Based Model** [a]
Any	1.69 (1.27–2.26)	1.78 (1.31–2.40)
Physical	1.83 (1.06–3.14)	1.52 (0.88–2.64)
Sensory	2.38 (1.56–3.63)	2.32 (1.52–3.52)
Cognition	1.69 (1.21–2.35)	1.77 (1.28–2.44)
Self-care	2.13 (0.93–4.88)	2.03 (0.86–4.78)
Independent living	1.55 (0.97–2.49)	1.56 (0.95–2.58)

[a] Models adjusted for confounder defined by the direct acyclic graph including age, race, and education.

Supplementary Table S4. Associations between disability and infertility-related doctor visit among those reported infertility.

Disability	Odds ratio (95% CI)	
	**Unadjusted**	**DAG-Based Model** [a]
Any	0.60 (0.30–1.20)	0.68 (0.32–1.44)
Physical	0.59 (0.29–1.20)	0.70 (0.31–1.60)
Sensory	0.60 (0.24–1.50)	0.54 (0.19–1.54)
Cognition	0.73 (0.33–1.62)	1.07 (0.46–2.49)
Self-care	1.65 (0.43–6.37)	2.07 (0.49–8.76)
Independent living	0.95 (0.44–2.08)	1.10 (0.52–2.33)

[a] Models adjusted for confounder defined by the direct acyclic graph including age, race, and education.

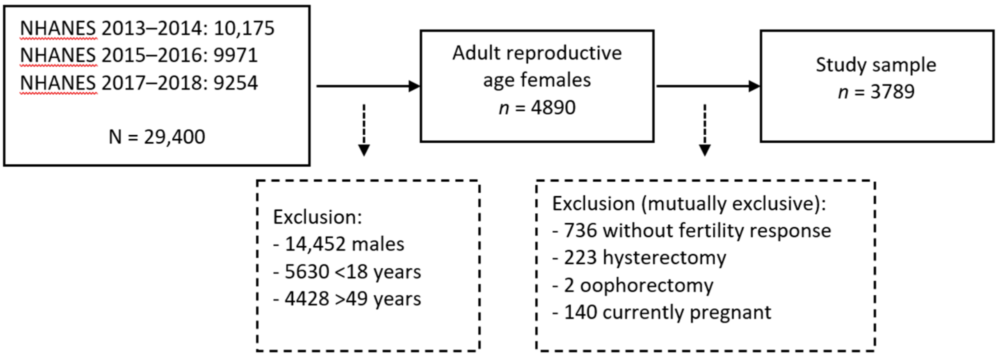

Figure S1. Study sample selection, National Health and Nutrition and Examination Survey (2013–2018).

A. Disabilities and infertility

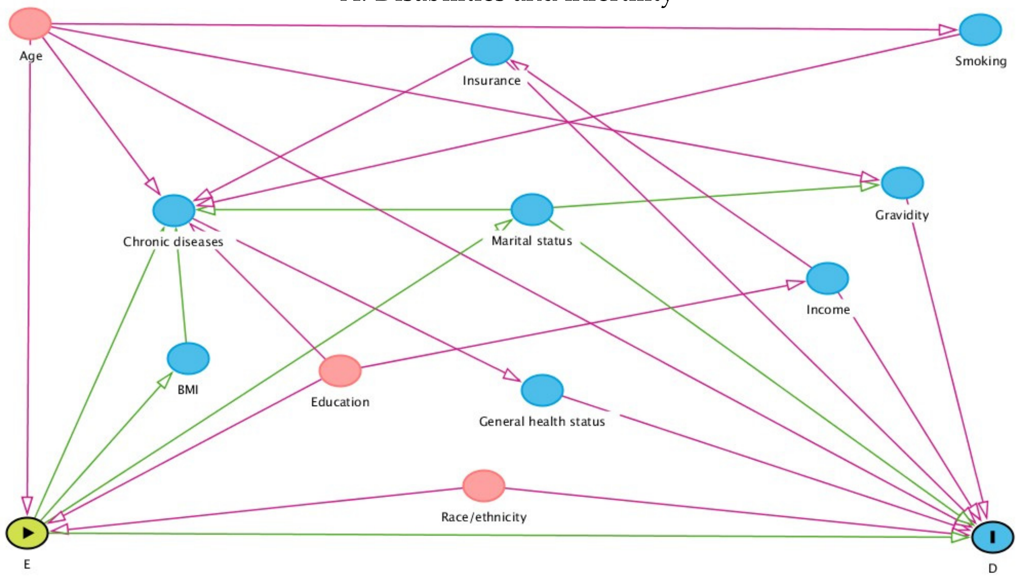

B. Disabilities and seeking infertility health care

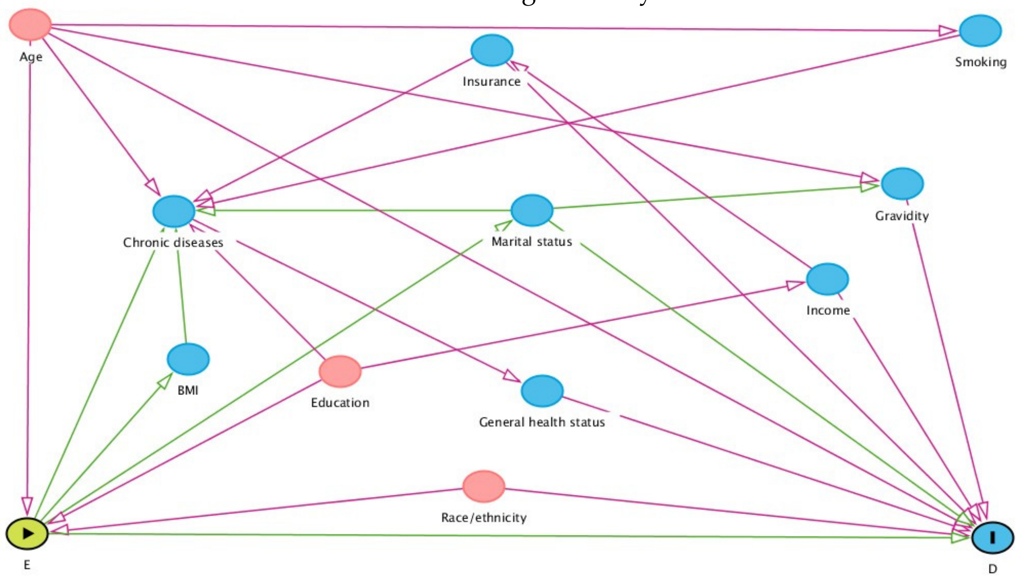

Figure S2. Direct acyclic graph to identify potential confounders for the relationship between (**A**) disabilities and infertility, and (**B**) disabilities and seeking infertility treatment. E—indicates exposure (independent variable). D—indicates disease outcome (dependent variable). Red circles—represent confounders. Blue variables—are not confounders but are involved in the causal relationship between exposures and outcomes. Abbreviations: BMI—body mass index.
